# Translation of Human β-Actin mRNA is Regulated by mTOR Pathway

**DOI:** 10.3390/genes10020096

**Published:** 2019-01-29

**Authors:** Irina Eliseeva, Maria Vasilieva, Lev P. Ovchinnikov

**Affiliations:** Institute of Protein Research, Russian Academy of Sciences, Pushchino, 142290 Moscow, Russia; mv.bioengineer@gmail.com (M.V.); ovchinn@vega.protres.ru (L.P.O.)

**Keywords:** translation regulation, mRNA, β-actin, ACTB, mTOR, amino acid starvation, 5’UTR

## Abstract

The mammalian target of rapamycin (mTOR) kinase is a well-known master regulator of growth-dependent gene expression in higher eukaryotes. Translation regulation is an important function of the mTORC1 pathway that controls the synthesis of many ribosomal proteins and translation factors. Housekeeping genes such as *β-actin* (*ACTB*) are widely used as negative control genes in studies of growth-dependent translation. Here we demonstrate that translation of both endogenous and reporter *ACTB* mRNA is inhibited in the presence of mTOR kinase inhibitor (Torin1) and under amino acid starvation. Notably, 5’UTR and promoter of *ACTB* are sufficient for the mTOR-dependent translational response, and the degree of mTOR-sensitivity of *ACTB* mRNA translation is cell type-dependent.

## 1. Introduction

The mTOR (mammalian target of rapamycin) signaling pathway is a well-known regulator of cell growth that responds to various growth stimuli, nutrients, energy, and stresses. As a master regulator of protein biosynthesis, the mTORC1 pathway is involved in cancer progression, obesity, diabetes, autism and aging [1,2,3]. Disturbance of the mTORC1 pathway is typical for various types of cancer, which makes mTOR one of the promising targets for chemotherapy [4]. Translation of mRNA of ribosomal and other translation-related proteins, such as elongation and initiation factors, is specifically reduced under mTOR inhibition [5,6]. This class of mRNA targets share a common TOP motif (terminal oligopyrimidine tract) located at the extreme 5’end and described as an uninterrupted stretch of 5–14 pyrimidines [7,8]. As shown recently, with inhibited cell growth, the translation initiation factor eIF4E can discriminate 2–3 nucleotides at the mRNA 5’ end [9]. Therefore, translation of TOP mRNAs is most sensitive to phosphorylation of 4EBP1 because when hypophosphorylated, the latter binds to eIF4E [10,11]. However, there are other mRNAs exhibiting growth-dependent translation inhibition, such as mRNAs with short 5’ UTRs carrying a specific TISU motif (translation initiator of short 5’UTR) [12] and some mRNAs with structured 5’ UTRs that are sensitive to RNA helicases [13,14]. Furthermore, growth-dependent translation regulation also involves eIF2 phosphorylation [15], codon optimality [16] and RNA-binding proteins [17,18,19].

*Glyceraldehyde 3-phosphate dehydrogenase* (*GAPDH*) [20,21,22,23,24] and *β-actin* (*ACTB*) [19,23,24,25,26,27,28,29] are the most popular negative control genes used in studies of growth-dependent translation. However, *GAPDH* was shown to be the mTOR target mRNA [16,30,31] carrying an almost canonical TOP [9,22,32,33]. *ACTB* is still used as the negative control [19,28], while changes of its mRNA translation are considered to be nonspecific and attributed to general consequences of mTOR inhibition.

Here we present an analysis of translation of endogenous *ACTB* mRNA and *ACTB* reporter mRNA under conditions of mTOR inhibition and amino acid and serum starvation. We report that *ACTB* mRNA exhibits growth-dependent translation whose inhibition in HEK293T and HeLa cells is comparable in magnitude with the canonical mTOR target mRNA of the ribosomal protein *RPL32*.

## 2. Materials and Methods

### 2.1. Plasmids

Promoter regions and 5’UTR were amplified from HEK293T genomic DNA. Genomic DNA was purified using GenElute Mammalian Genomic DNA Miniprep Kit (Merck KGaA, Darmstadt, Germany) following the manufacturer’s protocol. Sequences were amplified in the GC-rich buffer using Phusion High-Fidelity PCR Kit (Thermo Fisher Scientific, Waltham, MA, USA) and primers (Table S1). The primers had additional 20 nts per sequence for ligation-independent cloning (SLIC) into the pNL2.2 vector (Promega, Madison, WI, USA). Purified PCR products were cloned into the promoter-free pNL2.2 vector by SLIC. Briefly, 50 ng of amplified and DpnI-treated pNL2.2 was mixed with a fivefold molar excess of PCR product (promoter and 5’UTR). To obtain overlapping sticky-ends, the mixture was treated with T4-DNA polymerase (NEB, Upswich, MA, USA) in 1x NEBuffer 2.1 for 20 min at 22 °C. The reaction was stopped by addition of dCTP to the final concentration of 0.5 mM and incubation on ice for 5 min. To improve annealing, the mixture was incubated at 75 °C for 20 min and then at 50 °C for 10 min and transformed to *Escherichia coli*. The resulting constructs were named *pNL2.2 SLU7-NlucP*, *pNL2.2 ACTB-NlucP*, *pNL2.2 RPL32-NlucP*, *pNL2.2 YB-1-NlucP*.

### 2.2. Cell Cultures

HEK293T, HeLa, PC3 and SK-N-SH cells (originally obtained from ATCC, American Type Culture Collection) were kindly provided by Dr. Elena Nadezhdina (Institute of Protein Research, Russian Academy of Sciences, Pushchino, Russia). HEK293T cells were cultivated in DMEM (Dulbecco Modified Eagle Medium), HeLa and SK-N-SH cells—in DMEM/F12, and PC3 cells in RPMI (Roswell Park Memorial Institute Medium). The media were supplemented with 10% fetal bovine serum, 2 mM glutamine, 100 U/mL penicillin, and 100 μg streptomycin (PanEco, Moscow, Russia). The cells were kept at 37 °C in a humidified atmosphere containing 5% CO_2_. The cells were cultivated by standard methods. To inhibit the mTOR kinase, Torin1 (solved in DMSO, 500 nM; Tocris Bioscience, Bristol, UK), or DMSO (control) was added to the medium for 2 h. For amino acid and serum starvation, the cells were washed once with PBS then incubated for 2 h in Earle’s balanced salt solution (Thermo Fisher Scientific) supplemented with 1× MEM vitamin solution (Thermo Fisher Scientific).

### 2.3. Western Blotting

For Western blot analysis, the cells were rinsed two times with phosphate buffered saline and lysed in SDS electrophoresis sample buffer. Proteins were separated by SDS-PAGE and transferred onto a nitrocellulose membrane. The membrane was blocked for 1 h at room temperature with 5% nonfat milk in TBS (10 mM Tris-HCl, pH 7.6, and 150 mM NaCl) and incubated overnight at 4 °C in TBS-T (10 mM Tris-HCl, pH 7.6, and 150 mM NaCl, 0.05% Tween 20) supplemented with BSA (5%) and appropriate antibodies. The membrane was washed three times with TBS-T, incubated for 1 h with 5% nonfat milk in TBS-T and horseradish peroxidase conjugated goat anti-rabbit IgG (1:4000, 7074, Cell Signaling Technology, Danvers, MA, USA) and then washed three times with TBS-T. Immunocomplexes were detected using an ECL Prime kit (GE Healthcare, Chicago, IL, USA) according to the manufacturer’s recommendations. The rabbit monoclonal primary antibodies were from Abcam (ACTB, ab8227, Cambridge, UK) and Cell Signaling Technology: phospho-eIF2 S51 (D9G8), phospho-p70 S6 Kinase T389 (108D2), phospho-S6 ribosomal protein S235/236 (D57.2.2E), S6 ribosomal protein (5G10), phospho-4EBP1 T37/46 (236B4), 4EBP1 (53H11), histone H3 (D1H2). Histone H3 antibodies were used as 1:10000 dilution, other primary antibodies were used as 1:2000 dilution.

### 2.4. DNA Transfection

The cells were plated onto a six-well (or 10 cm) dish 24 h prior to transfection. The cells were transfected using Lipofectamine 3000 (Thermo Fisher Scientific). For transfection, 400 ng (2.4 μg per 10 cm dish) of background plasmid *pcDNA3.1HA* [14] and 200 ng (1.2 μg per 10 cm dish) of *pNL2.2* plasmid were incubated with 1.2 μl (6.2 μl per 10 cm dish) of P3000 and 1.8 μl (10.8 μl per 10 cm dish) of Lipofectamine 3000 in 100 μl (600 μl per 10 cm dish) Opti-MEM (Thermo) for 15 min and then added to the growth medium. 4–6 h later, the cells were plated onto a 48-well dish (NLucP activity or RNA analysis) or onto a new 10 cm dish (polysome analysis) and cultivated for 16–20 h prior to the experiment. For each particular reporter, we performed transfection in a single dish and then plated the transfected cells onto smaller dishes to avoid transfection efficiency bias, which were then used for technical replicates of test and control conditions. Transfection of different reporters was performed simultaneously.

### 2.5. NLucP half-life Time Measurement and Luciferase Assay

For half-life time measurements, the cells were cultivated in normal conditions or in the presence of Torin1 or under amino acid and serum starvation for 2 h. Then cycloheximide (0.1 mg/mL) was added, and the cells were additionally incubated for 0, 15, 30, 60, 90 min. After incubation with cycloheximide, the cells were lysed and luciferase activities were measured.

NlucP activity was measured using Nano-Glo Luciferase Assay System (Promega). Cultured cells were lysed with passive lysis buffer (PLB, Promega) for 15 min at 37 °C. Enzymatic activities of NanoLuc luciferase (NlucP) were assayed using GloMax 20/20 Luminometer (Promega). All transfections were repeated several times in different cell passages.

### 2.6. Polysome Analysis

Cells (typically 70% confluent cells per 10 cm dish) were collected in ice-cold PBS + cycloheximide (0.1 mg/mL), rinsed once with ice-cold PBS + cycloheximide (0.1 mg/mL) and lysed in 250 μl of polysome buffer (15 mM Tris-HCl (pH 7.6), 15 mM MgCl_2_, 300 mM NaCl, 1% Triton X-100, 0.1 mg/mL cycloheximide). Lysates were passed through a 26G needle, incubated on ice for 10 min, and centrifuged to remove cell debris at 4 °C at 12,000 g for 15 min. Lysates were loaded onto a linear 15–45% sucrose gradient (15 mM Tris-HCl (pH 7.6), 5 mM MgCl_2_, 100 mM NaCl, 0.01 mg/mL cycloheximide) and fractionated by ultracentrifugation in a SW-60 rotor (Beckman Coulter, Brea, CA, USA) of Oplima L-90K Ultracentrifuge (Beckman Coulter) at 45,000 rpm at 4 °C for 1 h. The sucrose gradients were divided into 16 fractions of 250 μl each. Fractions corresponding to polysomes (including mRNAs loaded with two or more ribosomes) and subpolysomes (including monosomes, ribosomal subunits, and mRNP) were united, and 10 ng of in vitro transcribed *firefly luciferase* (*fluc*) mRNA was added to each fraction for normalization. RNA was extracted from each fraction and analyzed by qPCR.

### 2.7. RNA Isolation, cDNA Synthesis and In Vitro Transcription

Cells for RNA isolation were collected in ice-cold PBS. To each sample 2.5 ng of in vitro transcribed *fluc* mRNA was added as an internal control. RNA from cells or gradient fractions was isolated using TRIzol LS (Thermo Fisher Scientific) according to the manufacturer’s manual. Total RNA was treated with dsDNase, and cDNAs were synthesized using Maxima H Minus First Strand cDNA Synthesis Kit (Thermo Fisher Scientific) according to the manual.

*Fluc* cap(+) polyA(+) mRNA was transcribed by SP6 RNA polymerase from *pSP36T-5’UTRYB1-Fluc-3’UTRYB1A50* linearized with HpaI and capped using a ScriptCap m7G Capping System (CellScript, Madison, WI, USA) as described previously [34].

### 2.8. 5’RACE

cDNAs for the 5’RACE analysis were synthesized using a Mint RACE cDNA amplification set (Evrogen, Moscow, Russia) according to the manufacturer’s recommendations. PlugOligo adapter and oligodT18 (Table S1) were used for cDNA synthesis. The first round of PCR was performed using NlucP- and PlugOligo-specific primers (Table S1) carrying additional Illumina adaptor sequences. PCR products were purified using Agencourt AMPure XP (Beckman Coulter) according to the manufacturer’s recommendations. The second round of PCR was performed using primers from NEBNext Dual Index Primers Set 1 for Illumina (NEB). PCR products were purified using AMPure XP and sequenced on a NextSeq (Illumina, San Diego, CA, USA) platform. The resulting reads were processed with cutadapt v. 1.18 [35] to remove adapter sequences and 5’ poly-G tracks produced by Mint reverse transcriptase. The read mapping to *pNL2.2 ACTB-NlucP*, *pNL2.2 RPL32-NlucP* sequences was performed with bowtie 1.1.1 [36]. The cumulative coverage by 5’ read ends was computed using bedtools v 2.27.0 [37]. The total number of reads mapped within the windows surrounding the transcription start sites was no less than 700 for *ACTB* and 900 for *RPL32*.

### 2.9. qPCR

Transcript abundance was determined by quantitative PCR (qPCR) using a DTlite Real-Time PCR System (DNA Technology, Moscow, Russia), qPCRmix-HS SYBR+LowROX (Evrogen), and primers specific for each transcript (Table S1). The efficiency of each primer pair was determined by a series of dilutions. Transcript abundance data were normalized to the abundance of *fluc* mRNA.

### 2.10. Statistical Analysis

We performed two-tailed Student’s *t*-test to determine the significance of the difference between NlucP activities or mRNA levels as indicated in Figure Legends.

## 3. Results

### 3.1. Translation of Reporter mRNA with Promoter and 5’ UTR of ACTB is mTOR-Sensitive in HEK293T Cells

**Figure 1:**
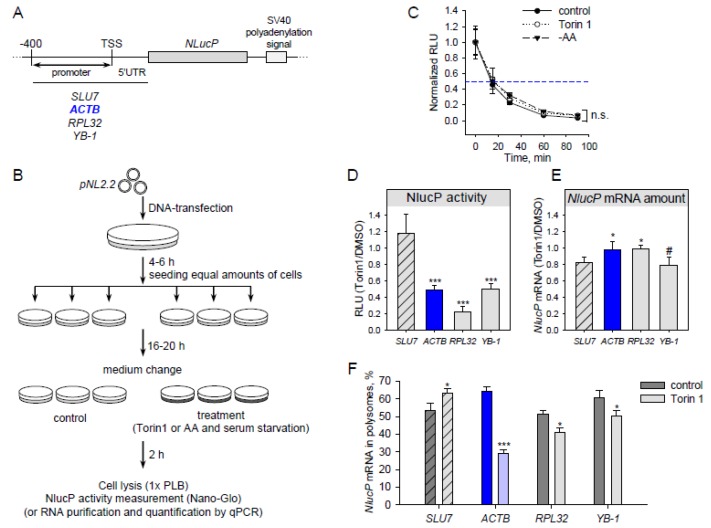
Translation of reporter mRNA with *β-actin* 5’UTR is sensitive to mTOR (mammalian target of rapamycin) inhibition. (**A**) Scheme of the *pNL2.2* plasmid used in this study (TSS—transcription start site). (**B**) Scheme of an experiment with *pNL2.2*. Cells were transfected with *pNL2.2*; after 4-6 h equal amounts of cells with the same transfection were seeded to smaller dishes to grow for 16-20 h. Cells were treated for 2h with DMSO (control) or 500 nM Torin1 or subjected to amino acid starvation (EBSS—Earle’s balanced salt solution). Two hours later, luciferase activity or mRNA abundance was measured. (**C**) HEK293T cells were transfected with *pNL2.2-SLU7-NlucP*; 24 h later the cells were treated with DMSO (control) or 500 nM Torin1 or EBSS (-AA) for 2 h, then CHX was added for 0, 15, 30, 60, or 90 min. After that, luciferase activity was measured. For each condition, the luciferase activity (*Y*-axis) was normalized to the value at the zero min time point. (**D**–**F**) HEK293T cells were transfected with *pNL2.2*. After 2h cell treatment with DMSO (control) or 500 nM Torin1 they were collected and luciferase activity (**D**), *NlucP* mRNA abundance (**E**), and polysome distribution (**F**) were measured. A polysome fraction was calculated as (mRNA in polysome)/(mRNA in polysome + mRNA in subpolysome). Values are the means of at least three independent experiments. Error bars show standard deviations. Student’s *t*-test was used to estimate the statistical significance versus the SLU7 control (**D,E**) or versus control treatment **(C,F)**, *** *p* < 0.001; * *p* < 0.05; #, n.s., nonsignificant.

The 5’end is important for mTOR-dependent translation. Therefore, we used a reporter system with the promoter and 5’UTR (Figure 1A) to ensure that reporter mRNAs receive 5’ ends similar to those of native RNAs. As a reporter gene, instead of traditionally used *firefly luciferase* (half-life time ~3 h [38]), we used the short-living *Nano luciferase, NLucP* (half-life time ~15 min, Figure 1C). This allowed measuring differences in protein synthesis resulting from short-term treatments. The scheme of the experimental workflow is shown in Figure 1B. For promoter regions we took −400 bp regions upstream of the CAGE (cap analysis gene expression) peak maxima (HEK293 data, [32]) and the respective full-length 5’ UTRs for the following genes: *RPL32* and *YB-1* (mTOR translational targets), *ACTB*, and *SLU7* (pre-mRNA splicing factor, negative control, translation of *SLU7* mRNA does not change upon mTOR inhibition according to Ribo-Seq data [10,11]). 

Particular mTOR inhibitors are able to stabilize proteins [30]. To avoid such a case, we measured NLucP activity at various time points of the translation elongation arrest (cycloheximide treatment) in normal conditions and upon mTOR inhibition. The NlucP protein half-life time was about 15 min in all tested conditions (Figure 1С). 

Upon mTOR inhibition by Torin1, translation of *SLU7* mRNA remained unchanged (Figure 1D). Surprisingly, *ACTB* mRNA translation decreased after Torin1 treatment to a degree similar to that of well-known mTOR-sensitive mRNAs (*RPL32* and *YB-1*). This effect does not result from changes in reporter mRNA abundance (Figure 1E). To ensure that the reduction in luciferase luminescence is indeed a result of decreased translation, we analyzed the reporter mRNA distribution in sucrose gradient fractions in normal conditions and after Torin1 treatment (Figure 1F and Figure S1). In the latter case, the amount of reporter *ACTB* mRNA was lower in the polysome fraction. This result, together with decreasing NlucP activity, indicates that *ACTB* reporter mRNA translation is inhibited after Torin1 treatment.

### 3.2. Translation of Endogenous ACTB mRNA is Sensitive to Growth Stimulus in HEK293T Cells

**Figure 2:**
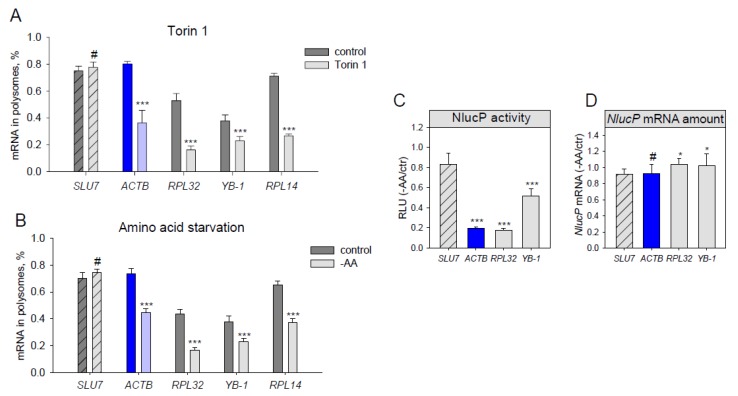
Translation of endogenous *β-actin* mRNA decreases under amino acid starvation and mTOR inhibition. (**A**–**B**) HEK293T cells were treated for 2h with DMSO (control), 500 nM Torin1 (**A**), or subjected to amino acid and serum starvation (**B**). The cells were lysed, fractionated at a sucrose gradient of 15–45%, and mRNA abundance of endogenous *SLU7*, *ACTB*, *RPL32*, *RPL14* and *YB-1* in polysome- and subpolysome fractions was measured by qPCR. A polysome fraction was estimated as (mRNA in polysome)/(mRNA in polysome + mRNA in subpolysome). (**C**–**D**) HEK293T cells were transfected with *pNL2.2*; after 2h of amino acid and serum starvation (-AA) the cells were collected, and luciferase activity (**C**) and *NlucP* mRNA abundance (**D**) were measured. Student’s *t*-test was used to estimate the statistical significance versus control treatment (**A**–**B**) or versus the *SLU7* control (**C**–**D**), *** *p* < 0.001; *p < 0.05; #—nonsignificant.

To further verify the result, we tested endogenous mRNAs in the same conditions as those for the reporter constructs. After Torin1 or DMSO treatment, we lysed the cells and analyzed the distribution of endogenous mRNAs in sucrose gradient fractions using qPCR (Figure 2A). In contrast to *SLU7*, the abundance of mRNAs of *ACTB*, *RPL32*, *RPL14*, and *YB-1* decreased in the polysome fraction after Torin1 treatment, with this effect being even more prominent as compared to the reporter constructs.

**Figure 3:**
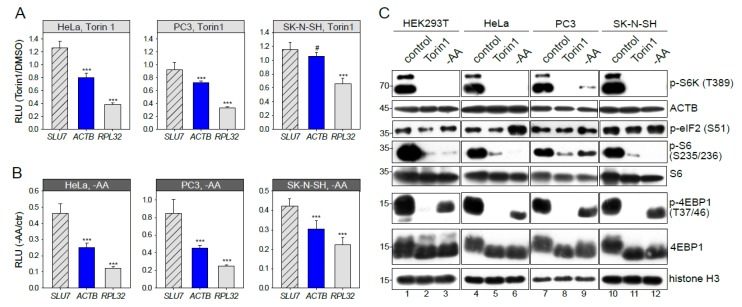
mTOR-sensitivity of *β-actin* mRNA translation is cell type-dependent. HeLa, PC3 or SK-N-SH cells were transfected with *pNL2.2*. After 2h of 500 nM Torin1 treatment (**A**) or amino acid and serum starvation, -AA (**B**) the cells were lysed, and luciferase activity was measured. Student’s *t*-test was used to estimate the statistical significance versus *SLU7*. (**C**) HEK293T, HeLa, PC3 or SK-N-SH cells were treated for 2h with DMSO (control), 500 nM Torin1, or subjected to amino acid and serum starvation (-AA). Cells were lysed and analyzed by Western blotting using the indicated antibodies. *** *p* < 0.001; #—nonsignificant.

Lastly, we checked if *ACTB* mRNA translation decreased not only due to direct chemical mTOR inhibition but also as a result of amino acid and serum starvation (which also leads to mTORC1 inhibition, Figure 3C). Translation of both endogenous (Figure 2B) and reporter (Figure 2С,D) *ACTB* mRNAs appeared to be inhibited to the degree similar to that of *RPL32*. Thus, we conclude that *ACTB* mRNA translation is growth-dependent.

### 3.3. mTOR-Sensitivity of ACTB mRNA Translation is Cell Type-Dependent

The *ACTB* mRNA was not recognized as an mTOR target in previous ribosome profiling studies [10,11] using other cell lines. Here we report that the response of *ACTB* translation to mTOR inhibition (Figure 3A) and amino acid and serum starvation (Figure 3B) is different in different cell types. Our experiments on HeLa and PC3 cells demonstrated the *ACTB* inhibition similar to that in HEK293T. However, in SK-N-SH cells, translation of reporter *ACTB* mRNA remained unchanged in the presence of Torin1 (Figure 3A). Quite the contrary, upon amino acid and serum starvation (Figure 3B), translation of *ACTB* mRNA visibly decreased, though to a lower degree than in the *RPL32* case. Thus, mTOR-sensitivity of *ACTB* mRNA translation is cell type-dependent.

The cell type-specificity of translational response could be attributed to a different degree of mTORC1 pathway inactivation upon growth inhibition. We analyzed the phosphorylation status of 4EBP1, S6 kinase, and S6 ribosomal protein (Figure 3C). As expected, phosphorylation of S6K, S6, and 4EBP1 was significantly decreased after Torin1 treatment and a bit less upon amino acid and serum starvation. Surprisingly, we did not find any major difference between the studied cell lines. Thus, the degree of mTORC1 pathway inactivation does not explain the cell type-specificity of translational response. Of note, we did not detect any decrease of the total ACTB protein level (Figure 3C). ACTB is an abundant and stable protein with an average protein half-life time of about 20 h [39] masking protein abundance changes in a limited time of 2h unless the protein is specifically degraded.

Next, we studied S51 phosphorylation of eIF2 (Figure 3C) which is known to be increased upon amino acid starvation. However, after 2 h, amino acid and serum starvation a significant increase of p-eIF2 (S51) was detected only in HeLa cells. This observation could explain the overall translational decline in HeLa upon amino acid and serum starvation but not the increased inhibition of *ACTB* and *RPL32* translation. Thus, the p-eIF2 (S51) is not a key factor of cell-type specificity either.

**Figure 4:**
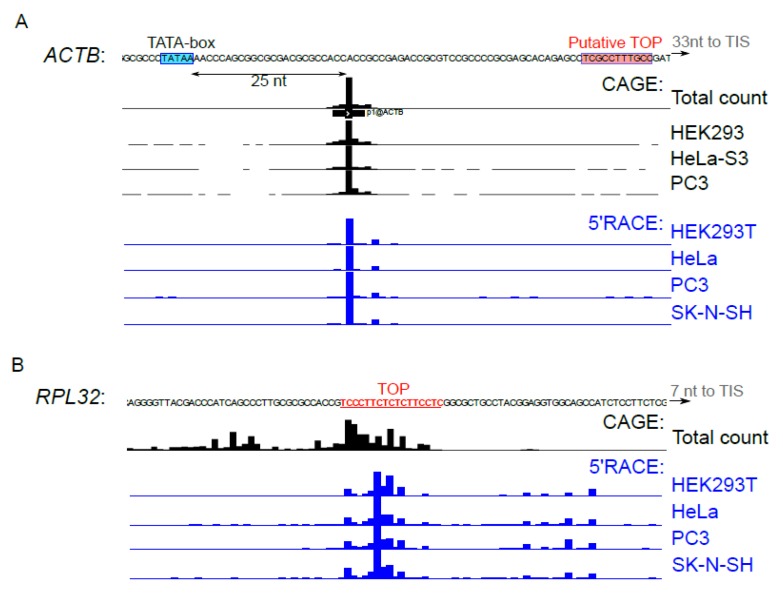
TSS activity in the studied cell types. (**A**–**B**) HEK293T, HeLa, PC3 or SK-N-SH cells were transfected with *pNL2.2-ACTB* (**A**) or *pNL2.2-RPL32* (**B**). After 24 h, the total RNA was extracted and 5’ends of the reporter mRNAs were analyzed by 5’RACE. HeliscopeCAGE signal [32] in the vicinity of *ACTB* or *RPL32* transcription start sites in HEK293, HeLa, PC3 cells and the average signal across all FANTOM5 cell types are presented. The TATA-box is shown in blue, putative TOP [22] (*ACTB*) and TOP (*RPL32*) are shown in red.

The activity of transcription start sites (TSS) differs from one cell line to another and can interfere with mTOR-dependent translational response [9,40,41]. We used 5’ RACE to determine the 5’ends of *ACTB* (Figure 4A) and *RPL32* (Figure 4B) reporter mRNAs in the studied cell lines. Distribution of the 5’ends of reporter mRNAs was almost identical in the tested cell lines and was in agreement with the CAGE data [32], for both *ACTB* and *RPL32*. Thus, cell-type specificity of *ACTB* translation mTOR response cannot be attributed to differences in regulatory elements of the cell type-specific 5’ UTRs.

All in all, mTOR-sensitivity of *ACTB* mRNA translation is cell type-specific. Nevertheless, this cell specificity cannot be attributed to alteration of 4EBP1 phosphorylation or altered TSS activity.

## 4. Discussion

Negative controls are crucial for the analysis of gene regulation. In growth-dependent translation studies, a typical approach is to use housekeeping genes such as *ACTB* or *GAPDH* [19,20,21,23,24,25,26,27,28,29,42,43]. In this study, we have shown that both endogenous and reporter *ACTB* mRNAs are sensitive to amino acid and serum starvation and to mTOR inhibition by Torin1. Of note, the changes of *ACTB* mRNA translation upon mTOR inhibition were observed in previous studies, but they were either ignored [44,45] or attributed to a nonspecific response [11,30,46]. Here we demonstrated that the magnitude of *ACTB* translational response is nearly the same as that for the canonical mTOR target *RPL32* mRNA. 

There were studies where *ACTB* mRNA translation did not respond to mTOR inhibition [19,23,24,25,26,27,28,29,47]. Probably, in some cases, this is connected with a particular experimental setup. Specifically, in [13,28,29] only three and more ribosomes were considered as a polysome fraction, which allowed identification of an additional set of mTOR-sensitive TISU-containing mRNAs. However, many canonical targets were missed, e.g., mRNA of ribosomal proteins. This probably can be explained by the fact that some of them mostly fall into light polysome fractions (2–3 Rs), at least in some cell lines [11,17,21,40,48]. In some cases, the difference can be attributed to cell type-specificity (Figure 3). This effect was observed even for canonical targets [49,50], which can result from the specificity of particular mTOR inhibitors [50], cell type-specific transcription start sites [9,40,41], different levels of expression of regulatory proteins, or activity of different branches of the mTOR signaling pathway [15].

Here we demonstrated that translation of reporter *ACTB* mRNA is mTOR-dependent and thus the respective regulatory element should be located within 5’UTR and/or promoter region. Notably, regulatory elements within promoter regions can influence translational response either by facilitating binding of specific RNA binding proteins to the nascent mRNA [51,52] or by dictating specific RNA modifications [53]. Thus, it cannot be ruled out that the promoter regions may be involved in the mTOR-dependent translational response.

The canonical mTOR target mRNAs of ribosomal proteins are transcribed from TCT-promoters whose functioning requires a pyrimidine-rich element that undergoes partial transcription and becomes incorporated in the 5’region of the incipient mRNA, thereby forming TOP [54]. Interestingly, a putative TOP was found for mouse *ACTB* mRNA [22], but it was not confirmed in large-scale studies [32,33]. Human *ACTB* promoter is a classical TATA-box promoter [32]; in the majority of cell types, it has a narrow transcription initiation region located about 25 nt downstream of the TATA-box (Figure 4A). The pyrimidine-rich sequence speculated to serve as TOP is located downstream in 5’UTR, and it is not clear whether it can participate in translational control.

Furthermore, regulation of growth-dependent translation of housekeeping genes can dramatically differ from that of ribosomal proteins probably not involving TOP at all. In particular, it was found that codon optimality can affect translational control in conditions of amino acid starvation, which is true for *ACTB* mRNA [16]. However, we detected mTOR sensitivity not only for endogenous but also for reporter mRNA lacking the *ACTB* coding region. Therefore, for *ACTB*, there should exist means of mTOR-dependent translational control other than codon optimality. Recently it was shown that (m^6^A) mRNA methylation determines translational efficiency and depends on the rate of transcription elongation [53]. Also, it was shown that the translation of many cellular mRNAs becomes mTOR-dependent upon N6-adenosine-methyltransferase catalytic subunit *METTL3* knockdown [55]. Interestingly, the primary mTOR targets such as ribosomal proteins are highly expressed mRNAs which are almost free of m^6^A methylation [56]. We hypothesize that mRNAs of housekeeping genes, including *ACTB*, are highly expressed, and hence only slightly methylated, thus resulting in mTOR-dependent translation. So, non-housekeeping and/or weakly expressed genes such as *SLU7* can serve as a better negative control.

In sum, we have demonstrated that translation of *ACTB* mRNA is inhibited by amino acid and serum starvation and mTOR inhibition. The 5’UTR and a promoter are sufficient for growth-dependent regulation. The translational response of *ACTB* mRNA varies from one cell type to another and is more prominent in conditions of amino acid and serum starvation as compared to the chemical mTOR inhibition.

## Supplementary Materials

The following are available online at http://www.mdpi.com/2073-4425/10/2/96/s1, Figure S1: Polysome profiles of HEK293T cells, Table S1: Primers for promoter cloning, qPCR and 5’ RACE.
