# Supplementary material for: Translation of Human β-Actin mRNA is Regulated by mTOR Pathway
Source: Genes (Basel). 2019 Jan 29;10(2):96. doi: 10.3390/genes10020096 (PMC6410274; doi:10.3390/genes10020096)
Supplement: Supplementary file 1 [file genes-10-00096-s001.pdf]

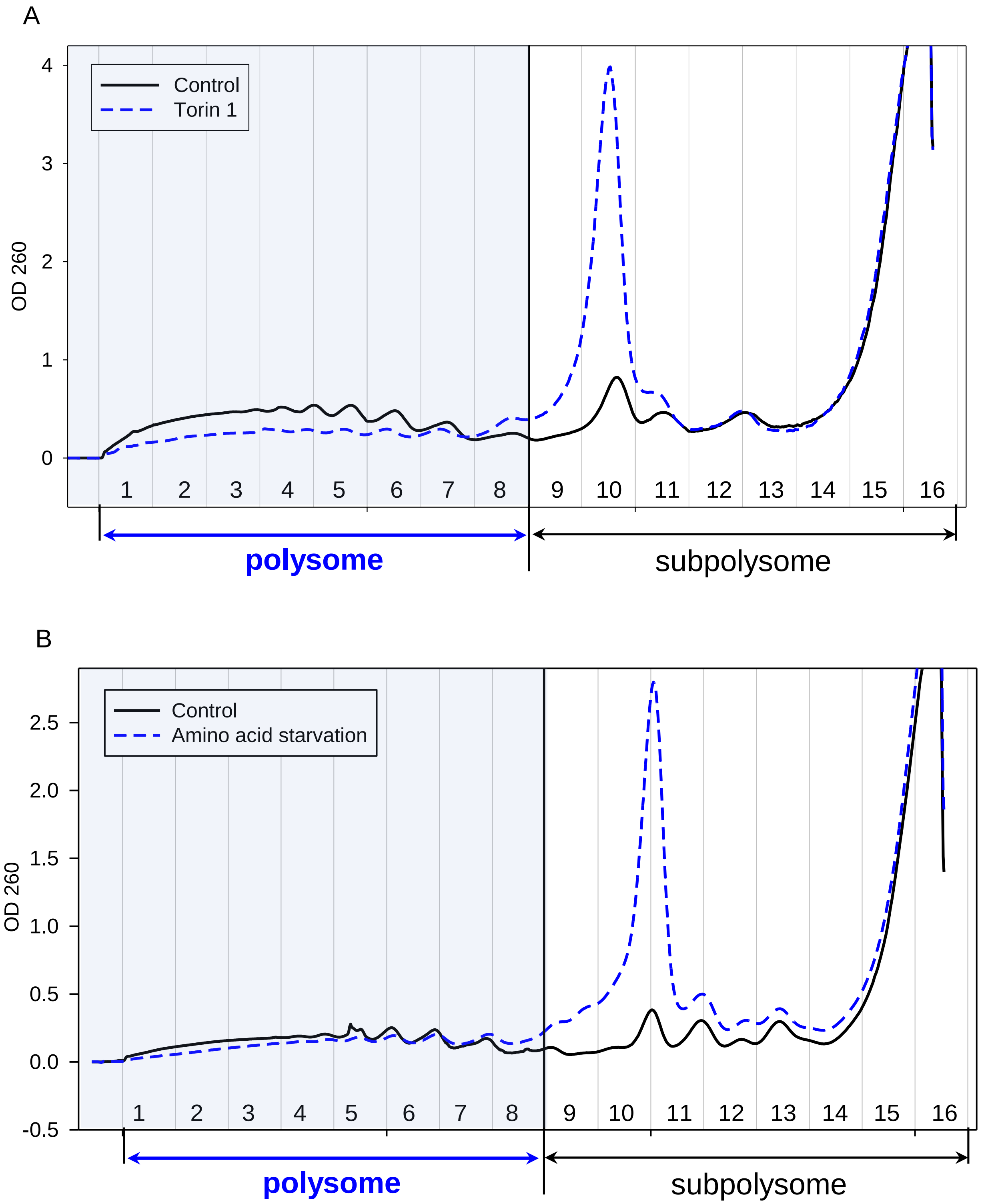

Supplementary Figure 1. Polysome profiles of HEK293T cells: untreated (control) and 2h treated with 500 nM Torin 1 (A) or 2h amino acid and serum starved (B). The cells were lysed and analyzed by sucrose gradient centrifugation. Typical profiles of optical density at A260 are shown. United polysome- (1-8) and subpolysome (9-16) fractions are marked.

Primers for cloning	
For_pNL2.2_1-20	ATGGTCTTCACACTCGAAGA
Rev_pNL2.2_4801-4820	AACAGTACCGGATTGCCAAG
For_ACTB_prom	CTTGGCAATCCGGTACTGTTCGCTCAGTGCCCAAGAGATGTC
Rev_ACTB_5UTR	TCTTCGAGTGTGAAGACCATGGTGAGCTGGCGGCGGGTGTGGAC
For_RPL32_prom	CTTGGCAATCCGGTACTGTTAGAAGCGTGGAGTTTGGAG
Rev_RPL32_5UTR	TCTTCGAGTGTGAAGACCATGATGCCGAGAAGGAGATGGCTGCCAC
For_SLU7_prom	CTTGGCAATCCGGTACTGTTCACAGCCTGGCTACTAATTAGTGCCAAGTTTAACTG
Rev_SLU7_5UTR	TCTTCGAGTGTGAAGACCATGGTTATCTGGCCTCCTCCAGCCCCGCCGCAGCTA
For_YB-1_prom	CTTGGCAATCCGGTACTGTTTCGAGCCCCCTTCCAATCC
Rev_YB-1_5UTR	TCTTCGAGTGTGAAGACCATGGTTGCGGTGATGGTG
**Primers for qPCR**	
For_fLuc	GGATTACCAGGGATTTCAGTCGATG
Rev_fLuc	GTTTTGTCACGATCAAAGGACTCTGGTAC
For_NLuc	GCTGTTCCGAGTAACCATCAAC
Rev_NLuc	GGTCCATACCGCTTTCTTGTG
For_ACTB	ACACCCTTTCTTGACAAAACCT
Rev_ACTB	CGCATCTCATATTTGGAATGACT
For_RPL14	AGCTCACTGATTTCATCCTCAAG
Rev_RPL14	TGTCATCTTGGCTTTCCTTTCTC
For_RPL32	TGCAACAAATCTTACTGTGCCGA
Rev_RPL32	TGGCATTGGGGTTGGTGACT
For_SLU7	TGATTTGGCAAAACGAACATTG
Rev_SLU7	TCCATCTGAGAATTTGGTTCCT
For_YB-1	AGGCGAAGGTTCCCACCTTA
Rev_YB-1	GTTGTCAGCACCCTCCATCA

Primers for cDNA synthesis (5'RACE)	
PlugOligo adapter	AAGCAGTGGTATCAACGCAGAGTACGGGGG
oligo-dT18	TTTTTTTTTTTTTTTTTT

Primers for 1st PCR round (5'RACE)	
For_RACE_PlugOligo	ACACTCTTTCCCTACACGACGCTCTTCCGATCTAAGCAGTGGTATCAACGCAGAGT
Rev_RACE_NlucP	GTGACTGGAGTTCAGACGTGTGCTCTTCCGATCTGGTCCATACCGCTTTCTTGTG

Supplementary Table S1. Primers for promoter cloning, qPCR and 5' RACE are shown. Sequences complementary to pNL2.2 and used for SLIC cloning are highlighted in red. Illumina adaptor sequences highlighted in blue.
